# Computational Model of Erratic Arrhythmias in a Cardiac Cell Network: The Role of Gap Junctions

**DOI:** 10.1371/journal.pone.0100288

**Published:** 2014-06-18

**Authors:** Aldo Casaleggio, Michael L. Hines, Michele Migliore

**Affiliations:** 1 Institute of Biophysics, National Research Council, Genova, Italy; 2 Dept. of Neurobiology, Yale University School of Medicine, New Haven, Connecticut, United States of America; 3 Institute of Biophysics, National Research Council, Palermo, Italy; University of California, Riverside, United States of America

## Abstract

Cardiac morbidity and mortality increases with the population age. To investigate the underlying pathological mechanisms, and suggest new ways to reduce clinical risks, computational approaches complementing experimental and clinical investigations are becoming more and more important. Here we explore the possible processes leading to the occasional onset and termination of the (usually) non-fatal arrhythmias widely observed in the heart. Using a computational model of a two-dimensional network of cardiac cells, we tested the hypothesis that an ischemia alters the properties of the gap junctions inside the ischemic area. In particular, in agreement with experimental findings, we assumed that an ischemic episode can alter the gap junctions of the affected cells by reducing their average conductance. We extended these changes to include random fluctuations with time, and modifications in the gap junction rectifying conductive properties of cells along the edges of the ischemic area. The results demonstrate how these alterations can qualitatively give an account of all the main types of non-fatal arrhythmia observed experimentally, and suggest how premature beats can be eliminated in three different ways: *a)* with a relatively small surgical procedure, *b)* with a pharmacological reduction of the rectifying conductive properties of the gap-junctions, and *c)* by pharmacologically decreasing the gap junction conductance. In conclusion, our model strongly supports the hypothesis that non-fatal arrhythmias can develop from post-ischemic alteration of the electrical connectivity in a relatively small area of the cardiac cell network, and suggests experimentally testable predictions on their possible treatments.

## Introduction

Understanding the basic cellular mechanisms underlying cardiac pathophysiology is of increasing importance, as the aging of the population predicts an increasing prevalence of cardiac morbidity and mortality. For this purpose, an important complement to experimental and clinical investigations is the mathematical modeling and simulation of the mechanisms responsible for cardiac electrophysiology [1], especially those that can underlie alterations in the propagation of electrical activity leading to arrhythmias. Although there are many types of arrhythmic cardiac behavior, most of the current models give emphasis to those with severe or fatal complications, such as atrial fibrillation [2] or ventricular fibrillation [3], and are based on the so-called reentry model. Reentry was first defined by Mines [4] as a persisting electrical impulse that reactivates an area of previously activated myocardial tissue that is no longer refractory, resulting in a circular movement of activation. The length of the circle depends on the impulse wavelength, defined as the product of the refractory period and conduction velocity (plus an excitable gap when present) [5]. The requirements for reentrant activation in the intact heart are a region of unidirectional block and a (regionally) slow-enough conduction velocity allowing an impulse to travel around or inside the affected region. The ultimate proof of reentry is its termination by interruption of the circle [4]. Our understanding of reentry has been extended by the introduction of different initiation mechanisms such as single rotor reentry [6], fibrillatory conduction [7]–[8], and the leading circle concept [9]. These mechanisms have been recently explored [3], cardiac tissue simulators have been presented [10], and the use of modeling in helping clinical practice has been suggested [11]–[13].

To explain the mechanisms underlying the initiation of a reentrant arrhythmic behavior, most models assume permanent changes in the intrinsic electrophysiological parameters of cardiac cells, such as altered intracellular Calcium dynamics [14] or ion channel modifications [15]. Other models consider alternative mechanisms, such as mitochondrial membrane potential oscillations and waves [16], or the roles played by individual sarcolemmal ion channels in atrial and ventricular fibrillation [17]. The major problem with these approaches is that the arrhythmic behavior is reproduced in an all-or-none fashion. In these models, the arrhythmia (usually a tachyarrhythmia) is often systematic, triggered by a single stimulus and, once initiated, does not stop spontaneously. This contrasts with what is observed in clinical practice, where thousands of relatively brief non-fatal episodes of arrhythmias occur during the life of a subject. Isolated premature ventricular beats (iPVB), bi- or tri-geminy sequences, couplets, triplets, and ventricular tachyarrhythmias (see the MIT-BIH Arrhythmia data base [18]), are commonly observed during the life of a subject without immediate life threatening conditions, although an increased rate of premature ventricular beats have been associated with an increased risk of sudden death in patients with heart failure [19]. Since these conditions are widespread in the population, it is important to investigate the malfunctioning mechanisms and how they can be treated.

In this paper, we explore with a computational model the possibility that non-fatal arrhythmias can originate from post-ischemic dynamic alteration of the electrical connectivity in a relatively small area of the cardiac cell network. The simulation findings show that random fluctuations of the intercellular gap junction conductance inside an ischemic area are sufficient to generate practically all of the observed types of transient arrhythmia. The model suggests possible treatments to reduce or eliminate these conditions.

## Methods

All simulations were carried out with the NEURON simulation environment (v7.3) [20] on a parallel BlueGene/Q IBM supercomputer (CINECA, Bologna, Italy). A typical 130 sec simulation required about 3 hours using 1024 processors. Model and simulation files will be available for public download on the ModelDB section of the Senselab suite (http://senselab.med.yale.edu/ModelDB/, acc.n.150691).

**Figure 1:**
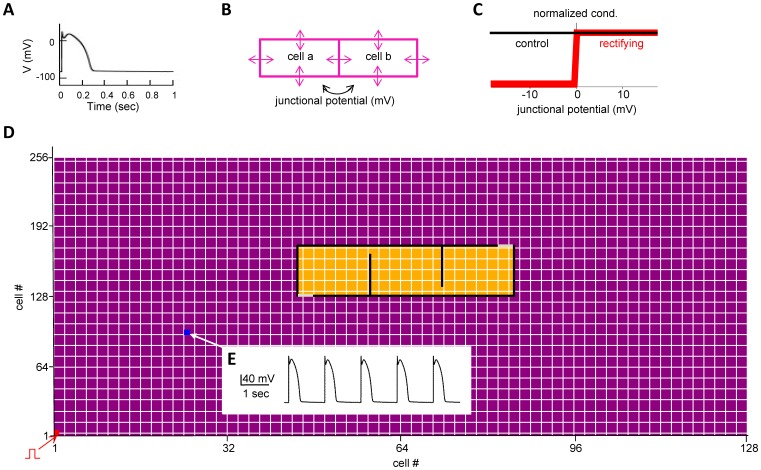
Model implementation. **A)** the action potential of an isolated cell in response to a short current pulse injection (4 nA, 5 ms); **B)** schematic representation of the gap junctions connecting neighbor cells, represented by arrows; **C)** the gap junction conductance as a function of the junctional potential under control conditions (black line) and for cells forming the edges of the fibrotic area (red line); **D)** Schematic representation of the full 128×256 cell network; individual cells are represented with small purple (normal) or yellow (ischemic) rectangles; the black lines identify the contour of the lesion, composed by non-conducting cells (i.e. g = 0). Cells with rectifying gap junctions, implementing entry and exit doors are shown in gray. Physiological input signals from Purkinje cells were modeled by periodically stimulating with a short current pulse cell(1,1) (bottom left of the network, shown in red); **E)** typical membrane potential of cell(25,100) under physiological conditions (i.e. no ischemic area).

We modeled a relatively small two-dimensional cardiac tissue of 12.8×4.1 mm, composed of 128×256 cardiac cells implemented as a single-compartment of 100×16 µm, corresponding to the real size of canine ventricular myocardial cells [21]. Electrophysiological passive and active properties were identical to those used in the Beeler-Reuter model [21], with model files downloaded from the public ModelDB database (http://senselab.med.yale.edu/modeldb, acc.n. 97863). This is one of the simplest realistic electrophysiology models for a single ventricular canine myocyte. It describes the cell activity on the basis of 4 trans-membrane currents: a sodium current, two potassium currents, and a calcium current which is responsible for the plateau potential occurring during a cell’s depolarization. A typical action potential generated in this cardiac cell model by a short (4 nA, 5 ms) current injection is shown in Fig. 1A. Each cell was connected via gap junctions with its 4 neighbors, as schematically represented in Fig. 1B. A gap junction between any two given cells under control conditions was modeled as a bidirectional, time-independent, ohmic conductance of 30 nS under control conditions, in agreement with experimental data [22] and within the wide range experimentally measured in mammals, which ranges from the 500 nS measured in ventricular pairs to the 8 nS in SA nodal pairs [23]–[24].

To model the abnormal conditions underlying different types of arrhythmias, we first implemented an ischemic area by altering the gap junction rectifying conductive properties (Fig. 1C) of the cells along the edges of the ischemic area (as illustrated in Fig. 1D). The gap junction conductance of the cells inside the area was reduced to a lower average value, which randomly fluctuated with time during a simulation (see Results). The complex behavior of gap junction conductance involved in cardiac function has been reviewed in Moreno [25]. Rectification of the gap junction conductance has been experimentally observed in HeLa cells [26]–[27], whereas fluctuations in the conductance during ischemia have been observed in dogs [28], rabbits [29] and humans [30]. This is the first time that the effects of time dependent fluctuations is investigated in the context of erratic cardiac arrhythmias.

The properties of an ischemic area were constrained by experimental observations. In particular, Peters et al. [31] found altered gap junctions as part of the early remodeling of myocardium after inducing infarction and ischemia in 6 dogs. Another study [32] proposed the existence of an entry and an exit door somewhere along the border of an ischemic region. We thus implemented a generic ischemic area as a region with propagation properties slower than normal tissue [33] (represented in yellow in Fig. 1D). The affected area was delimited by an almost completely closed contour which blocks signal propagation (black cells in Fig. 1D). Entry and exit doors were implemented along the contour with two small sections (grey cells in Fig. 1D) having strong rectification properties which allow only mono-directional communication between the normal external tissue and the ischemic inner region. Two different ischemic areas, of the same width (3.9 mm, corresponding to 39 cells) but different height (0.5–0.75 mm, corresponding to 30–45 cells) have been simulated.

During a typical 130 sec simulation, a pacemaker signal was generated by a periodic (every 800 ms) short current injection (4 nA, 5 ms) to cell (1,1) (indicated with a red marker in Fig. 1D). Gap junctions of cells belonging to the normal region were fixed to their control value of 30 nS, whereas the values of each gap junction inside the ischemic region were randomly chosen, every 500+/−50 ms, from a normal distribution with a given average and variance. Change the gap junction conductance to an average interval shorter than the normal periodic signal simplifies the analysis of the results. However, different intervals and mechanisms were also tested (see Results). Several combinations of gap conductance average value (range 4.5–5 nS) and variance (range 0.3–0.8 nS^2^) were tested. The membrane potential of cell(25,100) (indicated with a blue marker in Fig. 1D) under control conditions (i.e. no ischemic area) is shown in Fig. 1E. A movie illustrating the propagation of the activity following an external stimulation of cell(1,1) is reported in Movie S1.

It is important to stress that a number of additional mechanisms can be affected by an ischemic episode. Virtually all of them, from increased intracellular acidity [34] to changes in channel functioning [35], may independently contribute to the emergence of a PVB, and can lead to the generation of life-threatening arrhythmias. This is precisely why we did not include them in the model, at this stage. Rather, we were interested in studying the role, and isolating the effect, of random gap junction fluctuations. This is a process that is quite difficult to study experimentally. In this paper we have chosen to investigate only the functional consequences of an ischemic episode on gap junctions. It would be interesting to include the modulation of other mechanisms in a future study, to study how and to what extent they affect the basic findings shown in this paper.

### Comparison with Experimental Findings on Non-fatal Arrhythmias

**Figure 2:**
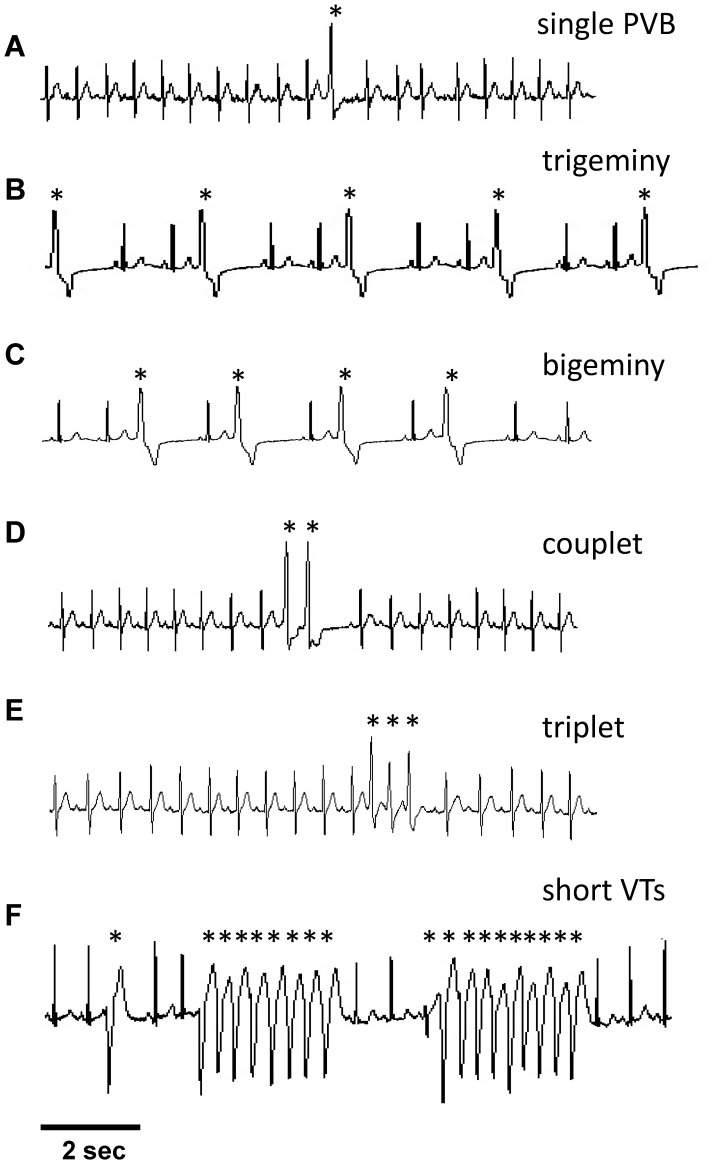
Typical experimental ECG recordings showing the different arrhythmias taken into account by our model. **A)** single premature ventricular beat; **B)** trigeminy complexes; **C)** bigeminy complexes; **D)** couplet episode; **E)** triplet episode; **F)** two short runs of tachyarrhythmias. In all cases, markers highlight arrhythmic complexes.

For a qualitative comparison of our model with experimental data we selected several representative electrocardiographic signals (ECG) from the Physionet Data Base [36] and shown in Fig. 2. In particular, we considered several 10 sec recordings from different patients with non-fatal arrhythmias commonly related to increasing cardiac electrical activity deterioration. These include: single premature ventricular beat (PVB), trigeminy sequences (a sequence of normal and premature beats with the ratio of 2∶1), bigeminy sequences (a sequence of normal and premature beats with the ratio of 1∶1), couplets (two consecutive premature beats), triplets (three consecutive premature beats) and, finally, a short run of non-sustained Ventricular Tachycardia (VT, a sequence of more than 3 premature beats that spontaneously recover to the normal condition).

## Results

**Figure 3:**
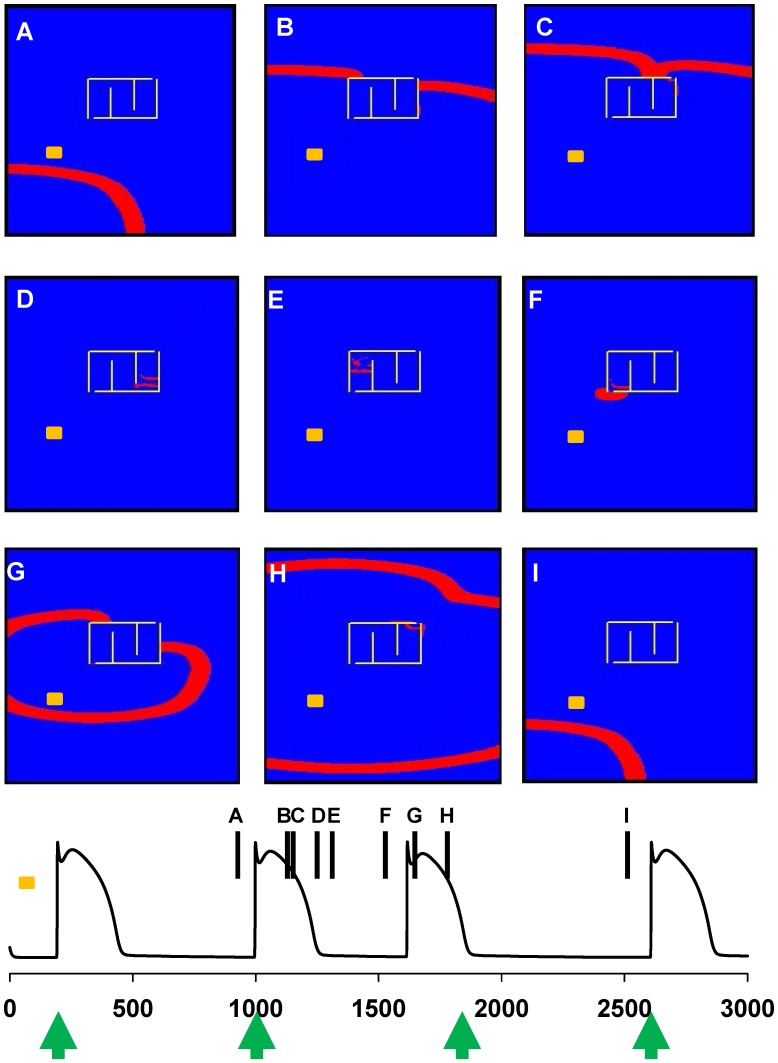
Typical onset of a premature beat. **A–I)** snapshots from Movie S2, illustrating the propagation of two normal beats (panels A-B-C and I) and a premature beat (panels D through H); *(bottom plot):* membrane potential of cell(25,100), marked in yellow in the snapshots, during the simulation shown in Movie S2. Vertical markers show the time points at which the snapshots in panels A-I were taken.

The main mechanism underlying the generation of premature beats suggested by our model is a direct consequence of the differential signal propagation inside an ischemic area. This process is illustrated in Fig. 3, where we show a few snapshots from Movie S2 and the membrane potential of cell(25,100) (indicated with a yellow mark in Fig. 3), outside the ischemic area. Under control conditions (i.e. without an ischemic area) each wave of activity generated by pacemaker cells will freely flow without interference (as shown in Movie S1). In the presence of a lesion (delimited by the yellow lines in Fig. 3), the signal propagation around the scar (see snapshots A-C and the corresponding time points in the bottom plot of Fig. 3) generates a secondary wave of activity inside the ischemic area (Fig. 3, snapshots D-E). Once it reaches the normal region (Fig. 3, snapshot F), it causes the generation of a premature beat. The activity spreads backward (Fig. 3, snapshots G-H) and negatively interferes with the generation of the expected beat in cell(1,1) (green arrow at t = 1800 ms in the bottom plot of Fig. 3), which is still within the refractory period. Activity returns to normal afterwards (Fig. 3, snapshot I). The arrhythmia does not occur every heart cycle because the gap junction random fluctuations do not allow reliable propagation of the signal inside the ischemic area (see Movie S2). These results show that an alteration in the gap junction conductance, caused for example by an ischemic episode in a relatively small area of cardiac tissue, can generate premature beats leading to arrhythmias.

**Figure 4:**
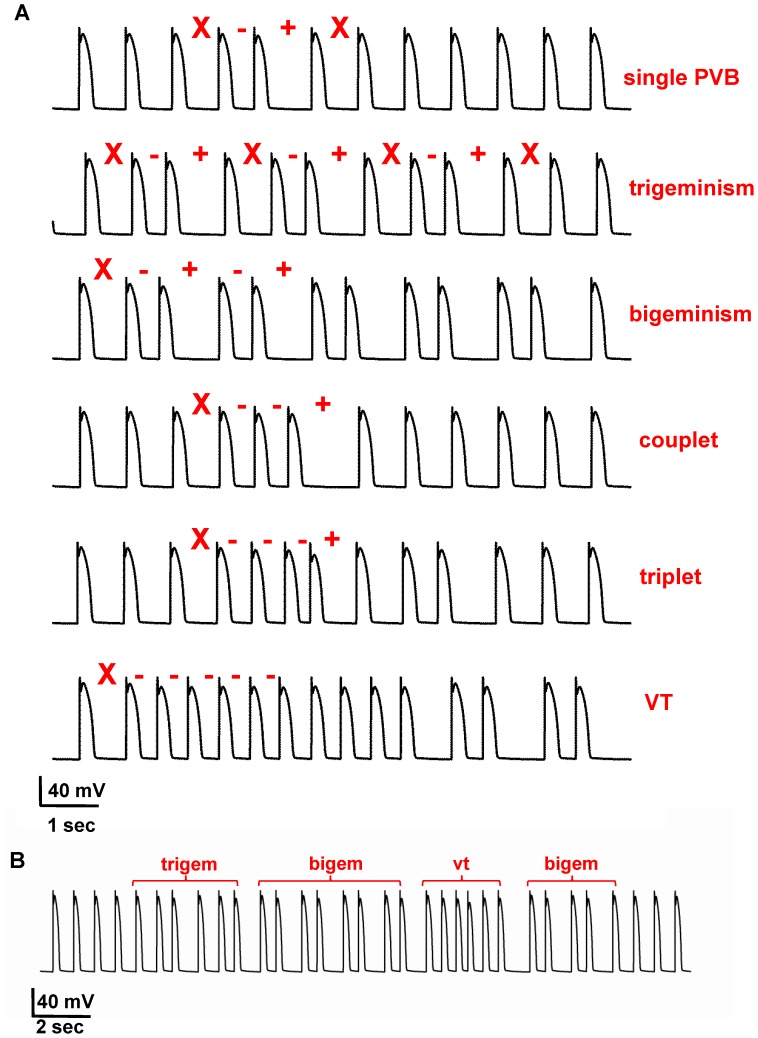
Our model suggests that all kinds of arrhythmia can be explained by dynamical fluctuations of the gap junctions. **A)** (top to bottom) isolated arrhythmia, trigeminy complex, bigeminy complex, couplet, triplet, short runs of tachyarrhythmias followed by a bigeminism; compare all panels with those in Fig. 2; **B)** 25 sec simulation exhibiting different types of arrhythmic behavior. In all cases, red markers highlight abnormal sequences (see Table 1); traces represent the membrane potential of cell(25,100), from simulations with the following average gap junction conductance and variance: (4.7, 0.3) iPVB, (4.9, 0.3) trigeminy, bigeminy, and triplet, (4.7, 0.6) couplet, (4.7, 0.8) VT.

To investigate this mechanism in greater detail, we carried out a systematic set of simulations using different values for the average and variance of the gap junction conductance inside the ischemic area. It should be stressed that, in all cases, the normal electrophysiological properties of all cells were not changed. Typical simulation findings exhibiting different kinds of arrhythmia are shown in Fig. 4A, where we plot selected excerpts of single cell recordings (cell 25,100) from simulations using different values for the average and variance of the gap junctions conductance in the ischemic area. The different types of arrhythmias where classified as shown in Table 1, by considering the sequence of interspike intervals having an abnormal duration with respect to that expected for normal cells. As can be seen, the model was able to qualitatively reproduce all types of experimentally observed arrhythmias (see Fig. 2). During each simulation we observed that, just as it occurs in the real system, different types of arrhythmias can appear at different times, and that their relative proportion depended on the average and variance of the gap conductance. A typical example is shown in Fig. 4B, where we show the membrane potential of cell(25,100) during a 25 sec time window of a simulation with a gap conductance of g = 4.7±0.7 nS. More or less organized premature beats appear throughout the simulation (labels above the trace in Fig. 4B). Taken together these results demonstrate that a single mechanism, namely the dynamical fluctuation of the gap junction conductance inside an ischemic area, is able to explain practically all kinds of non-fatal arrhythmias experimentally observed in cardiac cells.

**Table 1:** Specific sequences of interspike interval (ISI) define normal or abnormal behavior; X normal ISI; −, shorter than normal; +, longer than normal.

normal	X X X X X
single PVB	X −+X
trigeminism	X −+X −+X
bigeminism	X −+−+X
couplet	X − − X
triplet	X − − − X
vt	X − − − −

**Figure 5:**
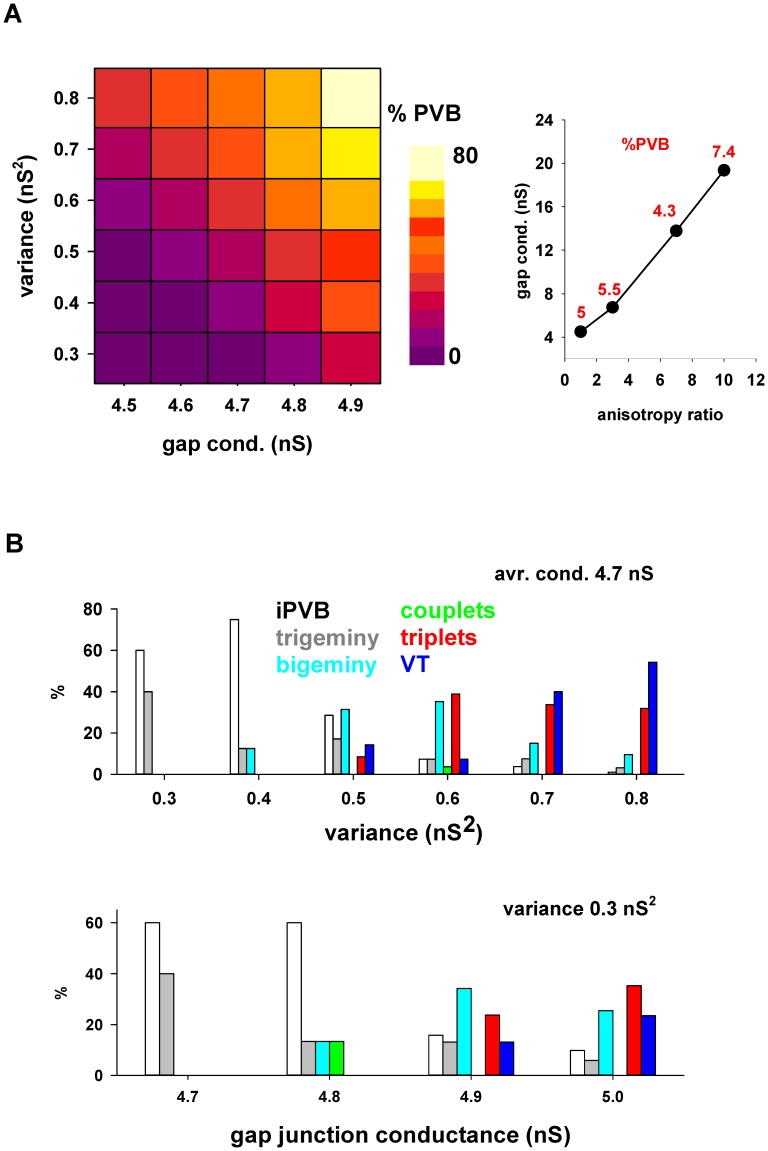
Gap junction modulations and fluctuations inside an ischemic area determine the relative proportion of premature beats. **A)** Percent of premature beats, with respect to the total number of beats, generated by different average and variance values of the gap junction conductance inside the ischemic area; **B)** Distribution of abnormal events as a function of the variance (top graph) or the average value (bottom graph) of the gap junctions conductance inside the ischemic area. White bars represent iPVB.

A more systematic exploration of the gap conductance parameter space is presented in Fig. 5A, where we report the proportion of PVB as a function of the average and variance of the fluctuations inside the ischemic area. The range of values for the gap conductance reproducing the arrhythmias is drastically lower than the value for normal cells. To the best of our knowledge, there are no direct measurements of the gap conductance within a cardiac ischemic area, except for the obvious case of dead cells (scars), which can safely be assumed to have a 0 conductance. Since one of the conditions that may affect this value is an anisotropic distribution of gap junctions on individual cells [37], we tested different anisotropy ratios (calculated as the ratio between the longitudinal and transversal gap conductance in a given cell). For this purpose, we started from a configuration with an average total gap conductance in each cell of 4×4.5 = 18 nS. An isotropic distribution resulted in 5% of PVB (Fig. 5A, left). As shown in Fig. 5A (right), a similar proportion of PVB (red labels in Fig. 5A, right) was obtained for increasing values of anisotropy with a corresponding increase in the average gap conductance. Interestingly, we found that the relative distribution of premature beats corresponding to the different kind of arrhythmias can be directly related to the variance of the gap conductance fluctuations. This is illustrated in the top plot of Fig. 5B, where we show that (for an average gap conductance of 4.7 nS) a progressively higher variance in the fluctuation results in a decrease of isolated premature beats (Fig. 5B, iPVB, white bars), and an increase of those involved with episodes of bigeminy, couplet, triplet, and tachycardia. The same effect, although less pronounced, was observed as a function of the average value (with a fixed variance of 0.3 nS^2^, bottom plot in Fig. 5B). These results suggest that random fluctuations in the gap conductance inside an ischemic area can promote and modulate the development of specific types of arrhythmic behavior.

**Figure 6:**
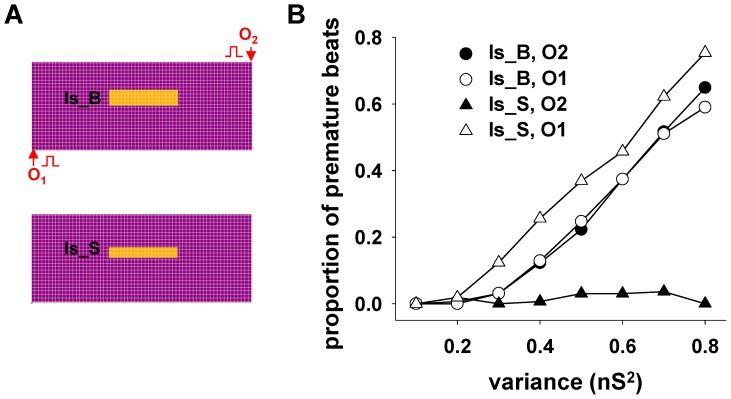
The generation of premature beats may depend on the direction of signal propagation. **A)** schematic representation of the two ischemic areas investigated, and the two stimulation points (O1 and O2) used to model different directions for the propagation of a physiological signal; **B)** Proportion of premature beats as function of the variance of the gap junction conductance inside the ischemic area for different stimulation points. The average value of the gap junction conductance of the cells inside the ischemic area was 4.7 nS in all cases.

To investigate the possible effects of ischemic areas of different sizes and positions with respect to the propagation of the normal electrical activity, we carried out a set of simulations using a different cell (O2 instead of O1, Fig. 6A) for the pacemaker stimulation. Simulations were also carried out using a 30% smaller area (Fig. 6A, Is_S), involving 1200 instead of 1800 cells. In these cases, the average value for the gap conductance was fixed at 4.7 nS. The results are shown in Figure 6B, where the fraction of premature beats is plotted as function of conductance variance. They suggest that when an ischemic episode creates a sufficiently large area of altered gap junctions (Fig. 6B, Is_B plots), the occurrence of premature beats does not depend on the direction of propagation of the physiological electrical activity. In contrast, a relatively smaller area (Fig. 6A, Is_S), may be much more sensitive to the propagation direction (Fig. 6B, Is_S). This occurs because the signal propagation delay inside a larger damaged area is sufficiently long to reach the normal region after the end of the refractory period of the normal tissue, independently from the origin of the external stimulation; for a smaller area (such as Is_S) this occurs when the signal arrives from O1 but not from O2. The phenomenon is illustrated in Movie S3 and Movie S4, respectively. Furthermore, consistent with the experimental findings of Woie et al. [38] showing that larger myocardial infarction areas lead to slower ventricular tachyarrhythmias, in our simulations the average interval during tachycardia events increased from 362±13 ms for the smaller Is_S case, to 545±21 ms for the larger Is_B.

To show the robustness of the results we carried out additional simulations. Starting from the typical configuration discussed in Fig. 3 we applied different variations, one at a time. We tested anisotropic gap junctions [37] concentrated at the ends (Movie S5) or at the sides (Movie S6) of the cells, 10% fluctuation of gap junctions in the entire tissue (Movie S7), and shorter (250 ms, Movie S8) or longer (1000 ms, Movie S9) average intervals for gap conductance fluctuations. One particularly intriguing possibility is for gap junction fluctuations to be an activity-dependent process. The implementation of a long- or short-term plasticity mechanism (analogous to what occurs in chemical synapses) was outside the scope of this work. However, as proof of principle, we ran an additional simulation in which gap fluctuations (4.7±0.5 nS) in each cell occurred on the rising phase of the action potential, corresponding to the heart contraction. We obtained a proportion of PVB similar to that obtained with the time-dependent fluctuations (19% vs 25%). Taken together these results demonstrate the robustness of our model under different conditions, with the emergence of a number of PVBs at random times in all cases.

Finally, assuming that ischemic areas can be modeled with regions of cells with malfunctioning gap junctions, we considered ways to reduce or eliminate premature beats and, thus, arrhythmias. Clinically, severe cases of ventricular arrhythmias are treated by the relatively minor surgical procedure of radiofrequency ablation [39], whereas quite extensive and invasive maze cuts are used for cases of atrial fibrillation [40].

Our model suggests that premature beats can be eliminated in three different ways: *a)* by closing the exit door from the ischemic region, as it is usually done at the hospital with the radio-frequency ablation procedure, *b)* by pharmacologically opening the entry/exit doors to the ischemic area, and *c)* by pharmacologically decreasing the gap junction conductance. To implement the surgical procedure (case *a*), we created additional lesions by setting to 0 the gap junction conductance of the cells surrounding the exit door of the ischemia. This precluded the propagation of an ischemic beat outside the ischemic area and prevented the generation of premature beats (see Movie S10). Case *b)* was implemented by strongly reducing the gap rectification property of the cells involved with the entry/exit doors, making them similar to those of normal cells. This practically removed the entry/exit doors, and had the effect of stopping the generation and propagation of abnormal beats outside the ischemic area (Movie S11). The remaining propagation of abnormal electrical activity inside the ischemic area (and the corresponding heart contractile activity) might explain why the infarction zone may appear relatively small during myocardial ischemia/reperfusion injury in open-chest dogs [41]. To implement case *c)*, we ran a simulation in which all gap junction conductances (including those of normal cells) were reduced by 10%. The results (Movie S12) show that the normal propagation of the signal was unaffected, but its propagation inside the ischemic area was severely hindered. A 10% change may seem relatively small to result in macroscopic effects, but it should be considered that the suggested pharmacological application would affect (by 10%) the entire population of gap junctions (i.e. normal and ischemic). This is sufficient (in our model) to affect the behavior of the signal propagation within the ischemic region (which is already barely able to propagate the signal) without interfering with the propagation in normal tissue. Of course this change may not be enough in the presence of larger ischemic regions. Taken together these results show that it could be possible to treat non-fatal arrhythmias using relatively minor surgical or pharmacological procedures.

## Discussion

The main aim of this paper was to explore the role of gap junction dynamical fluctuations in the occasional onset and termination of the (usually) non-fatal arrhythmias widely observed in the heart. In patients with ischemic cardiomyopathy, these events arise from an abnormal generation and propagation of electrical activity caused by more or less important ischemic episodes [42]. Because signal propagation in the heart occurs via gap-junctions mediating the interaction among neighbor cardiac cells, a deeper understanding of the functional consequences of gap-junction malfunctioning can be an important step to understand cardiac arrhythmias. In general, the dynamic reconfiguration of gap junction conductance can have non-trivial consequences in defining normal and pathological activity of a network of connected cells in the heart and also in the central nervous system [43].

With our model, we have demonstrated how the degradation of gap junctions inside an ischemic area is able to explain practically all kinds of non-fatal arrhythmias experimentally observed in cardiac cells. The average value and variability of the gap junction conductance can be directly related to the type and seriousness of the arrhythmic behavior, whereas their fluctuation with time determines the length of the episode. There is considerable experimental evidence for the role of gap junction conductance changes in cardiac arrhythmias. From a general point of view, gap junctions consist of transmembrane proteins called connexins, which assemble to form homomeric or heteromeric hemichannels (connexons) with an aqueous pore. In mammals, there are many types of connexins, and several of them (e.g. Connexin 40, Connexin 43, Connexin 45) are present in the cardiac tissue [25], [44]. The docking of two connexons leads to the establishment of a homotypic or heterotypic gap junction channel, according to the different possible combinations of connexins that can form a channel [25], [27]. For example, it has been shown that various pathological disorders can be associated with alterations in expression and modulation of connexin proteins [45]–[47]. In particular, in end-stage failing human hearts, Connexin43 expression is decreased, with respect to normal conditions, at both the mRNA and protein levels, due to both ischemic and dilated cardiomyopathy [48], and Connexin 40 plays a role in atrial fibrillation [49]–[50]. Furthermore, arrhythmogenic remodelling of activation and repolarization in the failing human heart has been associated with changes in the expression of connexins [51]. Finally, the atrial myocardium susceptible to atrial fibrillation can be distinguished from its non-susceptible counterpart by a reduced Connexin 40 expression [47].

A critical property suggested by our model, for the generation of premature beats, is the anisotropy of conductance in the cells forming the entry and exit doors to the ischemic area. Experimental evidence for this rectifying effect has been reported in studying the electrical properties of cells coupled by Connexin 40, Connexin 43 and Connexin 45 [25]–[27]. The post-ischemia formation of entry and exit doors, from an area with slower signal propagation properties, has been suggested by experimental findings [31]–[32]. The alteration of gap junction properties is also supported by a number of experimental findings and observations. For example, heterotypic gap-junction channels may exhibit rectification with respect to the junction potential [26], especially when the Connexons include Connexin 45 together with Connexin 40 or Connexin 43 [25], [27]. Also, ischemia has been associated with a reduced amount of active Connexin 43 [45], [52]–[53] and Connexin 40 [47]. Furthermore, the novel drug Rotigaptide has been shown to increase gap junction conductance [54] by increasing Connexin 43 activity [55]. Our model suggests that this drug is effective because, by increasing the fraction of phosphorylated Connexin 43, it contributes to the formation of homotypic gap junctions with symmetrical properties, rather than heterotypic gap junction. This mechanism can thus inhibit the formation of entry/exit doors.

Both gap junction alterations and the mechanism of re-entry have been previously explored with computational models to explain cardiac arrhythmic behavior [2]–[3], [14]. However, to take into account the initiation of arrhythmias, all these models assume a number of modifications to different model mechanisms. None of them take into account the spontaneous termination of the arrhythmic behavior. Different models for the human atrial fibrillation were reviewed and compared [2] and, in agreement with our study, the results suggest that a reduced conductance of the gap-junctions inside a damaged area may promote non-fatal arrhythmias. Another review [56] also focused on the atrial fibrillation and the role of re-entry, originating from the presence of an obstacle and correlated with the presence of a fibrotic area within the myocardium. In this case, two types of re-entry mechanisms were considered [32]: an inner loop, in which the ischemic region acts as a delay line of the front-wave, and an outer loop where the reentry originates around an obstacle. The outer loop [2], [56] has been shown to be a useful way to explain the initiation (but not the termination) of atrial and ventricular tachycardia or fibrillation. With our model we have shown that all types of non fatal arrhythmias and slow tachycardia can also initiate and terminate by assuming an inner loop as their origin.

The treatment of at least the most severe forms of arrhythmia is usually carried out with surgical procedures that can be quite invasive [39]–[40]. It is thus important to develop alternatives to reduce or eliminate the occurrence of more or less organized premature beats. Our model suggests a few experimentally testable predictions on the possible actions that, in principle, can be used: *a)* relatively minor surgery to close the exit door to the ischemic area (Movie S10), *b)* pharmacological actions to reduce or eliminate the rectification properties of the gap junctions (Movie S11) and, *c)* a relatively small (10%) pharmacological reduction of the gap junctions conductance (Movie S12). In all cases, the propagation of the activity inside the ischemic area would be hindered while propagation in the normal tissue remains essentially unaffected.

## Supporting Information

Movie S1 **Simulation under control conditions.**
(MP4)

Movie S2 **Simulation in the presence of an ischemic area (Is_B).**
(MP4)

Movie S3 **Simulation in the presence of an ischemic area of smaller size (Is_S) with origin in O1.**
(MP4)

Movie S4 **Simulation in the presence of an ischemic area of smaller size (Is_S) with origin in O2.**
(MP4)

Movie S5 **Simulation in the presence of an ischemic area and the total gap junction’s conductance for each cell distributed with 3∶1 ratio, between the ends and the sides of a cell, to represent a higher concentration of gap junctions at the ends of a cell.**
(MP4)

Movie S6 **Simulation in the presence of an ischemic area and the total gap junction’s conductance for each cell distributed with 1∶3 ratio, between the ends and the sides of a cell, to represent a higher concentration of gap junctions at the sides of a cell.**
(MP4)

Movie S7 **Simulation in the presence of an ischemic area, with all gap junction conductances independently undergoing an average 10% random fluctuation every 500±50 ms.**
(MP4)

Movie S8 **Simulation in the presence of an ischemic area, with all gap junction conductances independently undergoing an average 10% random fluctuation every 250±25 ms.**
(MP4)

Movie S9 **Simulation in the presence of an ischemic area, with all gap junction conductances independently undergoing an average 10% random fluctuation every 1000±100 ms.**
(MP4)

Movie S10 **Simulation in the presence of an ischemic area with ablation of the exit door.**
(MP4)

Movie S11 **Simulation in the presence of an ischemic area (Is_B) but without anisotropy of the gap junction’s conductance of the cells forming the entry/exit doors.**
(MP4)

Movie S12 **Simulation in the presence of an ischemic area (Is_B), but with all gap junctions conductance reduced by 10%.**
(MP4)
